# Human Herpesvirus 6A Induces Dendritic Cell Death and HMGB1 Release without Virus Replication

**DOI:** 10.3390/pathogens10010057

**Published:** 2021-01-11

**Authors:** Rasmus Gustafsson

**Affiliations:** Center for Molecular Medicine, Department of Clinical Neuroscience, Karolinska Institutet, 171 77 Stockholm, Sweden; rasmus.gustafsson@ki.se; Tel.: +46-70-6832982

**Keywords:** human herpesvirus 6A, HMGB1, dendritic cells

## Abstract

Human herpesvirus 6A (HHV-6A) is a common virus that has important immunomodulatory effects. Dendritic cells (DC) are key players in innate and adaptive immunity and are implicated in the pathogenesis of many human diseases, including infections. (1) Background: Previous studies have demonstrated suppressive effects of HHV-6A on key DC functions. (2) Methods: human monocyte derived dendritic cells were inoculated with HHV-6A and viral replication, cell viability, and release of high mobility group box 1 (HMGB1) protein from DC and of the cytokines IL-2, IL-4, IL-6, IL-10, TNF and IFN-γ after co-culture with allogenic CD4+ T cells were assessed. (3) Results: Nonproductive infection of HHV-6A in DC leads to titer-dependent cell death and the release of HMGB1 protein, and a Th2 polarization. (4) Conclusion: These immune responses aimed to clear the infection may also imply risks for inflammatory pathologies associated with HHV-6A such as multiple sclerosis.

## 1. Introduction

Human herpesvirus 6 (HHV-6) [1] is a ubiquitous virus that most individuals have been exposed to at the age of two years [2,3]. It is a member of the β-herpesvirus genus of the *Herpesviridae* family and as such can establish lifelong latent infections in the host. Since 2014, HHV-6 isolates are classified into two distinct virus species, HHV-6A and 6B [4]. Therefore, earlier studies often have not distinguished between these two viral species. In addition, as the viruses display a high degree of homology it has been difficult to discriminate between HHV-6A and 6B with diagnostic tests. HHV-6B is the causative agent of exanthema subitum [5], but no disease has been causally linked to HHV-6A. However, an accumulating body of data suggests an association between HHV-6A infection and multiple sclerosis (MS) [6,7,8,9], a multifactorial disease characterized by an over-active immune system and/or misdirected immune reaction.

High mobility group box 1 (HMBG)-1 is a highly conserved alarmin, or damage-associated molecular pattern molecules (DAMPs), with antiviral effects [10] located in the nucleus and is passively released upon cell death. It has several redox isoforms that regulate its pro-inflammatory activity in various conditions, both at sterile states and during infections [11]. For infectious states, HMGB1 is elevated during respiratory infections such as during Respiratory syncytial virus (RSV) bronchiolitis [12] and is released by herpes simplex virus (HSV)-2 infected epithelial cells upon cell death [13]. Furthermore, HMGB-1 release has been seen to aggravate Drug Reaction with Eosinophilia and Systemic Symptoms (DRESS) [14], a syndrome with confirmed involvement of HHV-6A/6B [15,16]. Thus, there is a possibility that HHV-6A may trigger HMGB-1 release. The aim of the present study has been to investigate this possibility, and the data suggest that replication independent exposure of human dendritic cells (DC) to HHV-6A indeed induces titer-dependent cell death, HMGB-1 release and skewing the CD4+ T cell population towards a T helper (Th) 2-profile upon co-culture with allogenic T cells.

## 2. Results

### 2.1. DC Do Not Support HHV-6A Viral Replication

**Figure 1:**
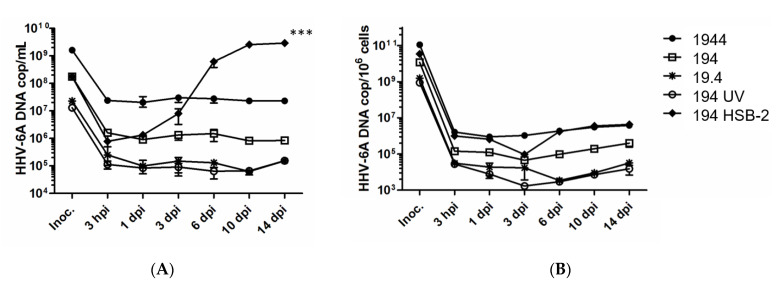
10^4^ dendritic cells (DC) were inoculated with 1944 to 19 TCID_50_ of human herpesvirus (HHV)-6A in untreated form, and with 194 TCID_50_ in UV-inactivated form. 10^4^ HSB-2 cells were inoculated with HHV-6A at 194 TCID_50_ as positive control for infection. The cells were inoculated for three hours before they were washed and incubated for 14 days. Virus replication was investigated by assessing accumulation of extracellular (**A**) and intracellular (**B**) HHV-6A DNA using Q-PCR analysis with primers and probe targeting the immediate early (IE) region of the HHV-6A genome. Data show representative results from one of three donors and the results are means ±SEM for three parallel cultures wells and analyzed using Single Factor ANOVA, *** *p* < 0.001.

To assess if DC can support HHV-6A replication, 10^4^ DC were inoculated with HHV-6A at 1944 to 19 tissue culture infective dose (TCID_50_), or with UV-treated virus (194 TCID_50_), used as negative control for infection. As positive controls for infection 10^4^ HSB-2 cells, which are known to support HHV-6A replication, were inoculated in parallel with 194 TCID_50_ HHV-6A. Quantitative polymerase chain reaction (Q-PCR) analysis of the HHV-6A DNA levels in cells and supernatants of DC cultures for up to 14 days after infection suggests that HHV-6A persists but does not replicate in DC at all titers tested (Figure 1A,B). This notion was further supported with data on UV-treated HHV-6A that gave a similarly flat growth curve. In contrast, for the HSB-2 cultures accumulation of viral DNA was seen over time in the supernatants (*p* < 0.001), suggesting that the virus inoculum was infectious. Intracellular increase of HHV-6A DNA was also seen, but as the HSB-2 cells continued to expand after inoculation the virus growth curve appears to be flat, given the unit HHV-6A DNA/cell. A cellular receptor for HHV-6A is CD46 [17], a molecule known to be expressed by all nucleated cells including DC. Therefore, DC are likely susceptible for viral entry and indeed in a previous report HHV-6A proteins were detected in moderate amounts in DC after inoculation and showed a similar staining pattern as did DC inoculated with UV-inactivated HHV-6A [18]. In contrast, HSB-2 inoculated with the same HHV-6A supernatants showed strong staining. These notions in combination with the data in the current report suggest that HHV-6A can enter but not replicate in human monocyte-derived DC.

### 2.2. Exposure of HHV-6A by DC Induces Virus Titer-Dependent Cell Death, HMGB1 Release and Th2 Skewing

**Figure 2:**
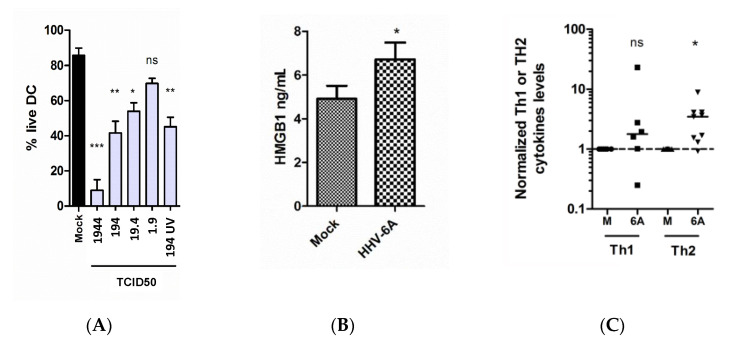
HHV-6A inoculation induces DC cell death (**A**), HMGB-1 secretion (**B**) and skewing towards a Th2 profile when co-culture with allogenic CD4+ T cells (**C**). In panel A, the bars represent the mean results (± SEM) of two to five donors. In panel B, the bars represent the mean results (± SEM) of four donors. In panel C, data points represent cytokine levels measured using cytometric bead arrays (CBA) of IL-2, IL-4, IL-6, IL-10 and IFN-γ in supernatants of co-cultures of DC and allogenic T cells. DC cultures from three individual donors were inoculated for three days with HHV-6A (6A) or mock (M) before they were co-cultured with allogenic T cells. The data points are normalized to mock exposed DC. IL-2 and IFN-γ represents the Th1 group and IL-4, IL-6 and IL-10 represents the Th2 group. Solid black lines represent median values for each group. The data were analyzed using t-tests. * *p* < 0.05, ** *p* < 0.01, *** *p* < 0.001, ns: not significant.

To investigate how HHV-6A exposure of human DC affects cell viability, 10^4^ DC were inoculated with 1944 to 1.9 TCID50 of HHV-6A, or with mock as negative control. Trypan blue staining and analysis using Bürken-chambers revealed that HHV-6A induces cell death in a virus titer-dependent manner (*p* < 0.001, *p* < 0.01, *p* < 0.05 or *p* > 0.05 compared to mock for 1944, 194, 19 or 1.9 TCID_50_, respectively) (Figure 2A). The data suggest that cell death seen for HHV-6A inoculated DC is independent on virus replication as DC inoculated with UV-treated HHV-6A at 194 TCID_50_ died to the same extent, as did DC inoculated with non-UV-treated HHV-6A, also at 194 TCID50 (*p* > 0.05 compared to mock).

To investigate if the DC cell death seen may induce an inflammatory response, HMGB1 release was determined in supernatants from cultures of 10^4^ DC inoculated with HHV-6A at 194 TCID_50_, or treated with mock, using ELISA targeting HMGB1. Increased concentrations of HMGB1 were seen in supernatants of HHV-6A inoculated DC, compared to in supernatants of mock treated DC (*p* < 0.05) and suggests that exposure of the virus indeed induces HMGB1 release (Figure 2B).

To investigate if HHV-6A exposure may affect the Th profile, supernatants of cultures of 10^4^ DC were inoculated with HHV-6A at 194 TCID_50_ for three days, or with mock, and co-cultured with allogenic CD4+ T cells. The co-cultures were harvested after four days and subjected to cytometric bead arrays (CBA) targeting IL-2, IL-4, IL-6, IL-10 and IFN-γ. While the expression of cytokines in the Th1 group (IL-2 and IFN-γ) did not differ between co-cultures of CD4+ T cells and DC treated with mock or HHV-6A, cytokines of the Th2 group (IL-4, IL-6 and IL-10) were significantly elevated in the co-cultures of CD4+ T cells and DC exposed to HHV-6A.

## 3. Discussion

DC are key immune cells that play important roles in capturing foreign antigens that enter the body, including viruses, and initiate specific immune responses against pathogens by presenting antigens to naïve T cells. However, DC can also be targets for viral infections, and with their antigen capturing capacity may be exposed to large amounts of virions. HHV-6A and 6B are very successful viruses that most people have been exposed to, as seen with a high seroprevalence in the human population [9] and have developed various specific viral immune-escape mechanisms that can hamper the ability of DC to activate T cells [19,20].

MS is recognized as an inflammatory disease [21] supported by large genetic studies that have linked several immune genes to the incidence of MS [22,23]. In addition, several environmental factors with well-established impacts on immunity have been associated to MS. These include vitamin D [24], which is important for T cell activation, and smoking, which has effects on both humoral and cell-mediated immunity [25]. As a central factor in innate immunity driving pro-inflammatory responses that can mediate autoimmunity, HMGB1 may be an important driver in MS [26]. Indeed, previous publications report data on HMGB1 expression in MS lesions [27] and elevated serum levels of HMGB1 in treatment of naïve MS patients, compared to those receiving disease-modifying treatment [28]. Even though an accumulating body of data suggests an association between HHV-6A infection and MS [6,7,8,9] the mechanism of action remains unclear. The data in the current study show that DC die upon exposure in a viral load dependent manner and release HMGB1, but this release is independent on virus replication. 

Thus, it is possible that HMGB1 release upon cell death induced by HHV-6A infection and/or reactivation may act as a driver in inflammation in MS, but more studies are needed to further investigate this notion, and to test whether this can happen in vivo. In addition, if HHV-6A exposure leads to passive release of HMGB1 simply due to cell death or whether the virus has impacts on HMGB1 expression remains to be investigated.

## 4. Materials and Methods

### 4.1. Isolation of Peripheral Blood Monocytes and Generation of DC

Buffy coats were bought from the Blood Transfusion Clinic at the Karolinska University Hospital, and monocytes were isolated from the buffy coats using commercial kits (RosetteSep Human Monocyte Enrichment Cocktail solution, Stemcell, Grenoble, France) and density gradient centrifugation on lymphoprep (Fresenius Kabi Norge AS, Oslo, Norway). To generate DC, the purified monocytes were cultured in RPMI 1640 medium containing GlutaMAX (Invitrogen Gibco, Paisly, UK) supplemented with 10% fetal bovine serum (HyClone,, Logan, UT, USA), 100 µg/mL streptomycin and 100 U/mL penicillin (Invitrogen Gibco), 250 ng/ml of recombinant human granulocyte-macrophage colony-stimulating factor (GM-CSF) (R&D systems or PeproTech, London, UK) and 6.5 ng/ml of recombinant human IL-4 (R&D systems). RPMI 1640 is described above, but without GM-CSF and IL-4 is referred to as complete RPMI, whereas RPMI 1640 with no supplementary agents is referred to as incomplete RPMI. On day 3 of culture, half of the medium was replaced with fresh complete RPMI containing IL-4 and GM-CSF. On day 6 or 7 the cells were harvested and washed with incomplete RPMI medium. The cell population used for the experiments described below typically contained >70% CD14− CD1a+ live cells, assessed by flow cytometry (data not shown).

### 4.2. HHV-6A Propagation and Inoculation

HHV-6A strain GS [1] was propagated in HSB-2 cells. When the cytopathic effect (CPE) was >50%, the cell culture was cleared by two rounds of centrifugation for 10 min at 300 g and the supernatant was collected and stored in aliquots at −80 °C. The 50% tissue culture infective dose (TCID_50_) was determined by ocular inspection for cytopathic effect as previously described [29] according to the Reed and Muench method. 10^4^ DC were incubated with mock (supernatant from uninfected HSB-2 cells) or HHV-6A at 1944, 194, 19.4 TCID_50_ for 3 h before they were washed with incomplete RPMI medium and cultured in complete RPMI supplemented with GM-CSF and IL-4 at 5% CO_2_ and 37°C. 10^4^ HSB-2 cells were inoculated in parallel with 194 TCID_50_ as positive controls for infection. DC were also incubated with HHV-6A supernatant that had been inactivated by UV irradiation for 20 min. This treatment completely eliminated the replication capacity as evident by TCID_50_ assays with HSB-2 cells as targets but as previously shown, uptake of UV-inactivated HHV-6A can be detected by confocal microscopy [18], suggesting that this treatment does not lead to breakdown of viral proteins. Assessment of DC viability was determined using Trypan blue staining and manual counting in Bürken chamber.

### 4.3. Assessment of HHV-6A Replication in DC

Viral replication was followed at 3 hours post-infection (hpi) and at 1, 3, 6, 10 and 14 days post-infection (dpi) in cells and supernatants by assessing viral DNA accumulation using Q-PCR (7500 Fast Real-Time PCR System, Applied Biosystems, Warrington, UK) of the immediate-early gene as previously described [30]. DNA was extracted from cells or supernatants using a commercial kit according to the manufacturer’s protocol (MagMAX-96 Viral RNA Isolation Kit, Applied Biosystems).

### 4.4. Assessment of HMGB1 Release

To assess if DC exposed to HHV-6A can release HMGB1, cell-free supernatants from cultures of 10^4^ DC inoculated with 194 TCID_50_ of HHV-6A and incubated for three days were collected and subjected to HMGB1 enzyme linked immunosorbent assay (ELISA) according to the instructions of the manufacturer (IBL International, Hamburg, Germany).

### 4.5. Co-Culture of DC and Allogenic T Cells and Cytokine Measurements

Peripheral blood mononuclear cells (PBMC) were isolated from buffy coats bought from the Blood Transfusion Clinic at the Karolinska University Hospital using density gradient centrifugation on lymphoprep (Fresenius Kabi Norge AS). CD3+ and CD4+ T cells were further isolated from these PBMC using magnetic micro bead kits (Miltenyi Biotech GmbH, Germany), according to the manufacturer’s instructions. Cell numbers were determined using a phase contrast microscope (Nikon) and Trypan blue (Gibco) staining. 3 × 10^4^ live DC and 10^5^ allogenic T cells per well were incubated for 96 hours at 5% CO2 and at 37 °C. Subsequently, supernatants from the co-cultures were harvested and subjected to cytometric bead arrays (CBA) (BD Biosciences, Franklin Lakes, NJ, USA) targeting the cytokines IL-2, IL-4, IL-6, IL-10, IFN-γ and TNF and analyzed with a Gallios flow cytometer (Beckman Coulter, Brea, CA, USA), and data were analyzed using the software FCAP (Soft Flow Inc., St. Louise Park, MN, USA).

### 4.6. Statistics

The data were analyzed in the GraphPad Prism v.5 software (GraphPad, La Jolla, CA, USA) using Student’s t-test with two-tailed p-values and 95% confidence interval and Excel (Microsoft, Redmond, Washington, USA) using Single Factor ANOVA with α 0.05.

## Data Availability

The data presented in this study are available on request from the corresponding author.
